# Electrospun fibrous sponge via short fiber for mimicking 3D ECM

**DOI:** 10.1186/s12951-021-00878-5

**Published:** 2021-05-08

**Authors:** Yan Li, Juan Wang, Dejian Qian, Liang Chen, Xiumei Mo, Lei Wang, Yan Wang, Wenguo Cui

**Affiliations:** 1 Department of Plastic Surgery, Shandong Provincial Maternal and Child Health Care Hospital, Cheeloo College of Medicine, Shandong University, Shandong, 250061 People’s Republic of China; 2 Department of Burn Surgery, Clinical Medicine, Weifang Medical University, Shandong, 261053 People’s Republic of China; 3 Department of Orthopaedics, Shanghai Key Laboratory for Prevention and Treatment of Bone and Joint Diseases, Shanghai Institute of Traumatology and Orthopaedics, Ruijin Hospital, Shanghai Jiao Tong University School of Medicine, 197 Ruijin 2nd Road, Shanghai, 200025 People’s Republic of China; 4 Department of Orthopedic Surgery, The First Affiliated Hospital of Shandong First Medical University & Shandong Provincial Qianfoshan Hospital, Shandong Key Laboratory of Rheumatic Disease and Translational Medical Shandong, 250014 Jinan, People’s Republic of China; 5 State Key Laboratory for Modification of Chemical Fibers and Polymer Materials College of Chemistry, Chemical Engineering and Biotechnology, Donghua University, Shanghai, 201620 China

**Keywords:** Extracellular matrix, Electrospun, Micro/nano fibers, Sponges, Tissue regeneration

## Abstract

**Background:**

Most of the natural extracellular matrix (ECM) is a three-dimensional (3D) network structure of micro/nanofibers for cell adhesion and growth of 3D. Electrospun fibers distinctive mimicked 2D ECM, however, it is impossible to simulate 3D ECM because of longitudinal collapse of continuous micro/nanofibers. Herein, 3D electrospun micro/nano-fibrous sponge was fabricated via electrospinning, homogenization, shaping and thermal crosslinking for 3D tissue regeneration of cells and vascular.

**Results:**

Fibrous sponge exhibited high porosity, water absorption and compression resilience and no chemical crosslinked agent was used in preparation process. In vitro studies showed that the 3D short fiber sponge provided an oxygen-rich environment for cell growth, which was conducive to the 3D proliferation and growth of HUVECs, stimulated the expression of VEGF, and well promoted the vascularization of HUVECs. In vivo studies showed that the 3D short fiber sponges had a good 3D adhesion to the chronic wound of diabetes in rats. Furthermore, 3D short fibrous sponges were better than 2D micro/nanofiber membranes in promoting the repair of diabetic full-thickness skin defects including wound healing, hair follicle regeneration, angiogenesis, collagen secretion.

**Conclusion:**

Therefore, electrospun short fibrous sponges are special candidates for mimicking the 3D ECM and promoting 3D regeneration of tissue.

**Graphic Abstract:**

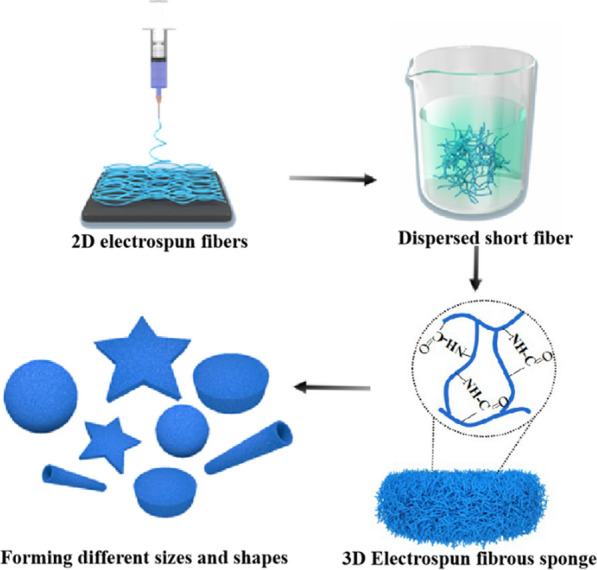

**Supplementary Information:**

The online version contains supplementary material available at 10.1186/s12951-021-00878-5.

## Background

Biomaterial scaffolds can mimic the structure of natural extracellular matrixes (ECMs) with such remarkable scale of accuracy such that they can actively participate and synergistically evolve with intrinsic cell activities [1]. Therefore, designing biomaterial scaffolds with structural morphology identical to that of the inherent three-dimensional (3D)-nanofibrous-network-like structure of ECMs is crucial for achieving optimal biomimetic performance [2, 3]. Nanofibrous scaffolds are characterized by high specific surface areas, which provide additional attachment points for cell adhesion and proliferation. Furthermore, their nanoscale fineness facilitates the precise structural remodeling of the microenvironment in which the cells live [4]. Recently, electrospinning has attracted notable attention in the field of tissue engineering, because of its capability of rearranging nano–micron-sized ultrafine fibers into specific scaffolds, whose morphology and structure resemble those of natural ECMs [5–7]. Numerous studies have demonstrated the capability of electrospun fibers (EFs) with varying compositions and structures to promote cell adhesion, proliferation, differentiation, and other related functions, by appropriately selecting the fiber materials, adjusting the electrospinning parameters, and/or modifying the surface of the electrospun nanofibers with bioactive molecules [8]. The ECMs in human tissue cells primarily exhibit 3D structures that provide a viable microenvironment for cell–cell interactions, migration, and survival [9]. Conventional EFs are mostly composed of densely packed fiber layers, however, because of their small pore sizes, low porosity, and lack of practical 3D structures, the 3D growth of cells along with the regeneration of specific tissues is limited [10, 11]. Thus, high-porosity and stereoscopic scaffold structures are prerequisites for ensuring an adequate nutrient exchange during cell growth and tissue reconstruction [12]. In this light, designing a 3D porous electrospun sponge-like bionic scaffold with high porosity, high water absorption, and excellent elasticity, is the primary goal of researchers in the field of tissue engineering.

In the recent decades, researchers have devised numerous novel strategies for reshaping EF membranes into 3D scaffolds, including gas foaming, liquid and template-assisted electrospinning, and multilayered electrospinning [13–16]. However, each technology has its drawbacks and limitations. For instance, an innovative 3D scaffold was fabricated with a 2D electrospun membrane by gas-foaming technology [17]. Although this approach effectively improved the shapes and pore sizes of the scaffolds to some extent, it cannot be implemented to realize 3D scaffolds with complex geometric shapes. Yokoyama et al*.* prepared 3D scaffolds by electrospinning in an organic solvent, however, their thickness was approximately < 3 mm [18]. Sun et al. directly developed an inflexible 3D scaffold by adjusting the collection template, but the aperture of the scaffold was small [19]. Unfortunately, although the aperture and morphology of the electrospun membrane can be adequately improved through the above-mentioned methods, the inability to precisely control the shape and pore size of the scaffold creates severe bottlenecks in the design of functional 3D scaffolds. Consequently, the aforementioned complications have prompted researchers to consider a method for remodeling the electrospinning-derived morphology of the scaffold, while preserving its original structure.

Recently, Ding et al. combined electrospinning and freeze-drying technologies to fabricate an ultra-light, porous aerogel composed of polyacrylonitrile (PAN) fibers, which demonstrated considerable potential for applications in oil–water separation and sound insulation, among others [11]. Si et al. fabricated a hyperelastic nanofibrous aerogel with a layered cellular structure [20], and Fong et al. reported a polycaprolactone (PCL) 3D scaffold [21]. In general, the methods discussed above present certain similarities: first, the EF membrane was crushed or ground into small pieces; subsequently, the small pieces were dispersed in the lyophilized liquid, freeze-dried, and finally cross-linked to obtain the stable form of the nanofiber scaffolds/aerogels. Compared with the conventional approach of using one technology, the combination of electrospinning and freeze-drying technologies for the fabrication of 3D scaffolds presents considerable advantages; nevertheless, certain disadvantages are present: (1) Study has shown that 3D scaffolds with pore sizes in the range of 100–300 μm can promote cell proliferation, migration and differentiation [22]. However, these methods usually generate scaffolds with relatively small pore sizes up to = 10–25 μm, which are not conducive to cell proliferation and infiltration; (2) the length of the short fibers is usually in the centimeter range, inducing the formation of a rough scaffold surface, which is not favorable for the regeneration of some types of tissues; (3) the fabrication process is typically complicated, as it requires the grinding of the nanofiber membrane in liquid nitrogen, limiting the shape control of the resulting scaffold and its preparation on a mass scale. To overcome these limitations, the combined use of electrospun micro- or nanoscale fibers with natural sponge structures will provide an effective method for realizing functional fiber scaffold systems for tissue engineering applications.

**Scheme 1:**
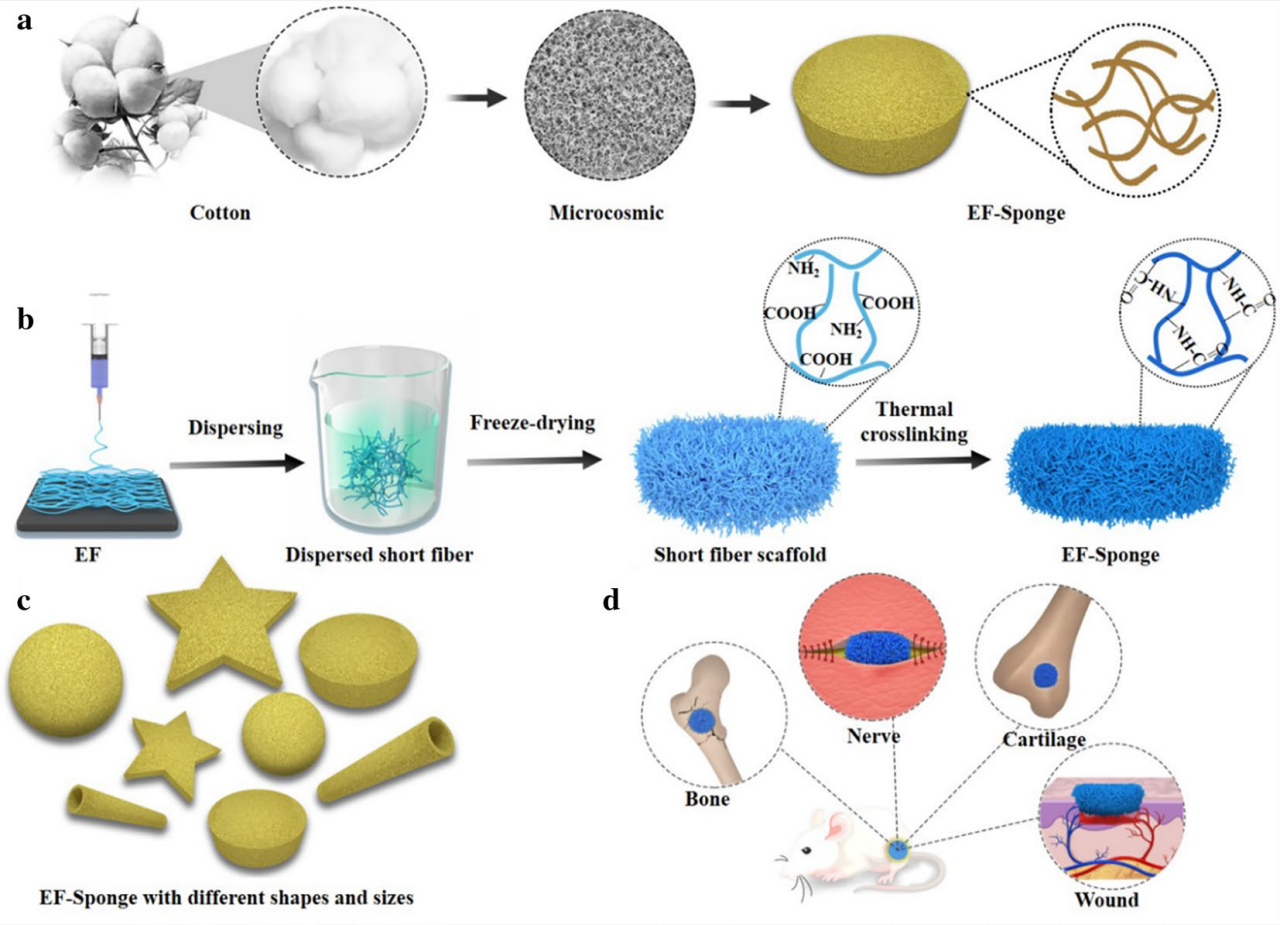
Schematic diagram of fabricating electrospun short fiber sponge scaffolds. **a** Unique biological structures of cotton fiber; **b** Schematic illustration of the fabricated process of EF-sponge scaffold; **c** EF-sponge of different sizes and shapes; **d** Application of EF-sponge scaffolds in different biological fields

In contrast to ordinary phloem fibers, cotton seed fibers are formed by the epidermal cells of fertilized ovules that undergo elongation and thickening. At an advanced stage during their development, cellulose is deposited on the cell walls and causes natural distortion, resulting in the porous structure of the cotton (Scheme 1a) [23, 24]. Herein, inspired by these unique natural biological structures, electrospun fibrous sponge (EF-Sponge) scaffolds with 3D morphologies and ECM biomimetic properties were fabricated using a novel and facile method-combination of electrospinning, high-speed homogenization, and thermal crosslinking technologies (Scheme 1b). No chemical crosslinked agent is used in the whole preparation process, and sponges are characterized by good water absorption and excellent shape-memory functionalities and can be produced in specific shapes and sizes to match the desired requirements (Scheme 1c). Firstly, the EF membranes were assembled into EF-sponge scaffolds with different fiber densities, and their differences in regards to water absorption and mechanical properties were compared. Secondly, in vitro the effects of our fabricated EF-sponge on the morphology, proliferation, and growth rate of human umbilical vein endothelial cells (HUVECs) were studied. Compared 2D electrospun fibers, the 3D sponge scaffolds were revealed to exhibit better in vivo 3D adhesion to the chronic wounds of diabetic rats, illustrating that the scaffolds effectively accelerated healing by absorbing the wound exudate, while stimulating the regeneration of collagen epithelium and generating new blood vessels during the process (Scheme 1d).

## Results and discussion

### The fabrication of scaffolds

**Fig. 1:**
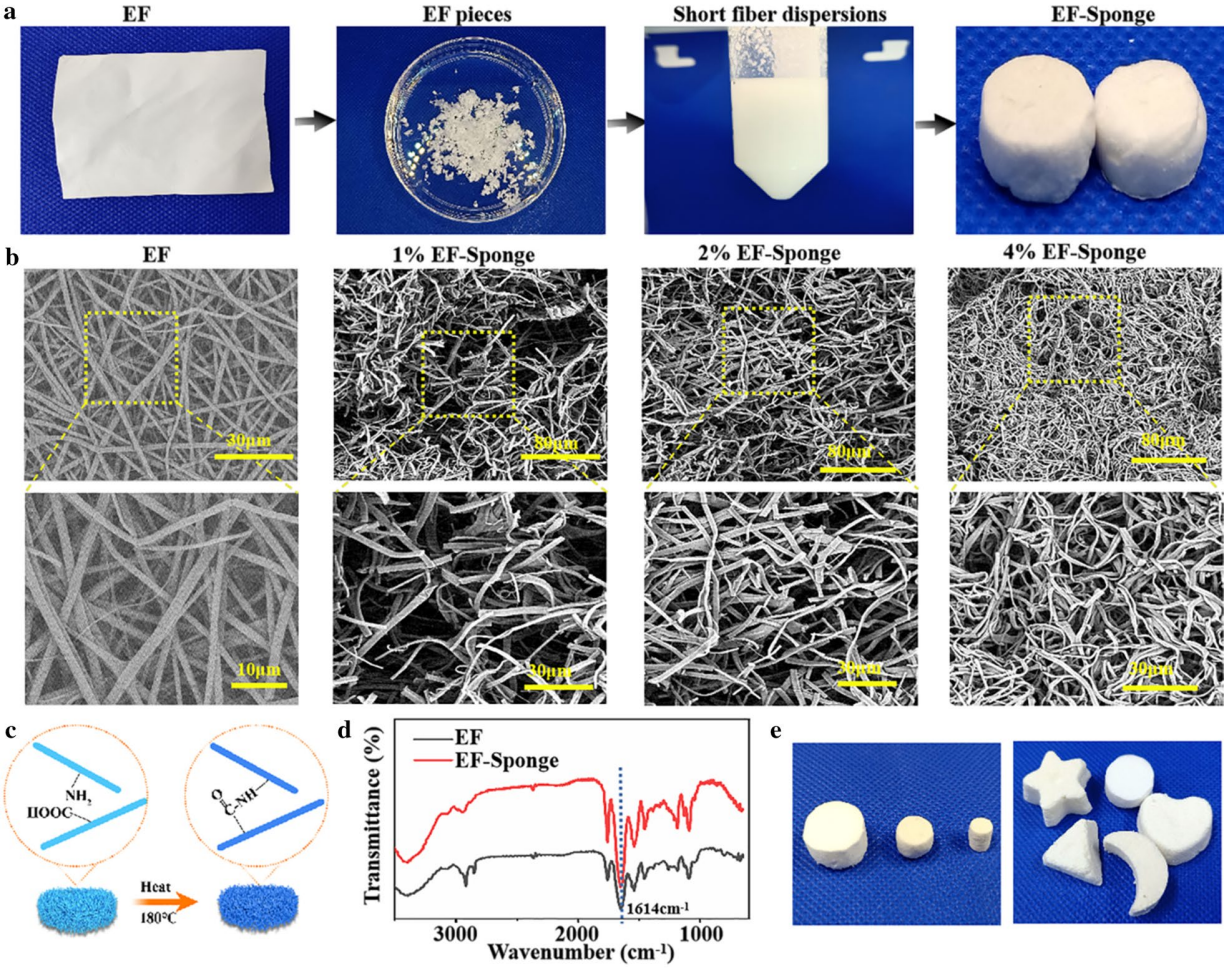
Characterization and morphology of EFs and EF-Sponges. **a** Schematic illustration of the fabrication of EF-Sponges; **b** SEM images of EFs and EF-Sponges with different fiber densities; **c** The schematic of thermal crosslinking mechanism; **d** FTIR spectra of EF and EF-Sponge; **e** Photograph of EF-sponges in different sizes and shapes

The schematic of electrospun short fiber sponge scaffolds fabrication was illustrated in Scheme 1b. It was divided into four steps: electrospinning, homogenization, shaping, and crosslinking. Initially, a gelatin/polylactic acid (PLA) solution (12 wt%) was prepared with a weight ratio of 4:1. Subsequently, gelatin/PLA fibers were prepared using the ordinary electrospinning equipment under suitable conditions, producing fiber membranes with relatively random coverage patterns. The fibers were cut into small fragments and dispersed in a tert-butanol solution by high-speed centrifugation, to ensure the uniform dispersion of the fibers (Fig. 1a). The dispersions were molded and freeze-dried to obtain the uncrosslinked scaffolds. Due to the hydrophilic nature of the gelatin base material, the scaffolds would quickly disintegrate when exposed to water. Therefore, crosslinking of scaffold is necessary and important to form a stable morphology and interconnected networks. To retain the original morphology of nanofibers, chemical reagents were often used for cross-linking [25]. However, chemical crosslinking agents are generally toxic for cell growth and tissue regeneration. Herein, to maintain high biocompatibility of the scaffold, the structure and morphology of the scaffolds were stabilized by thermal crosslinking, and no chemical crosslinking agent was used in the whole preparation process. After heating for 2 h, the structure of the EF-sponge maintained its stability even under intense mechanical agitation (Additional file 1: Video 1). Furthermore, no visible damage or physical alteration was caused because of the externally applied forces, confirming the good shape-memory functionality of the scaffold, and the scaffolds of varying sizes and shapes were attainable by altering the molds (Fig. 1e and Additional file 2: Video 2). Therefore, it was expected that 3D short fibers would demonstrate optimal applicability in the field of human tissue repair (Scheme 1d).

### Morphology and FTIR spectra of scaffolds

In order to investigate the differences in the fiber morphology and fiber density distribution of EF-sponge scaffolds, scanning electron microscopy (SEM) were performed. According to the results, the EFs had a randomly oriented non-woven-fabric-like morphology, with an average diameter of 910 nm. After breaking the EF membrane by using a high-speed homogenization technology, it was observed that the fiber diameters of the EF and EF-sponge samples were same, with an average short fiber length of ~ 27 μm, while exhibiting random fiber distribution. Moreover, the short-fiber density in the EF-sponge sample was discovered to vary with the solid content, i.e., as the solid content increased, the fiber density increased and the sponge structure became dense. More importantly, all scaffolds exhibited fibrous structures, with high resemblance to the natural ECM structures (Fig. 1b). Figure 1c illustrated the thermal crosslinking schematic diagram of the EF-sponge scaffolds. The heat treatment reduced the number of free acids and basic residues in the gelatin, which formed interchain crosslinks and amide bonds after thermal crosslinking. Fourier transform infrared (FTIR) analysis revealed that the absorption intensity peak (1614 cm^−1^) in the spectra of EF-sponge, which corresponded to the C − N stretching vibration band, was significantly stronger than that of EF (Fig. 1d).

### Water absorption capacity of scaffolds

**Fig. 2:**
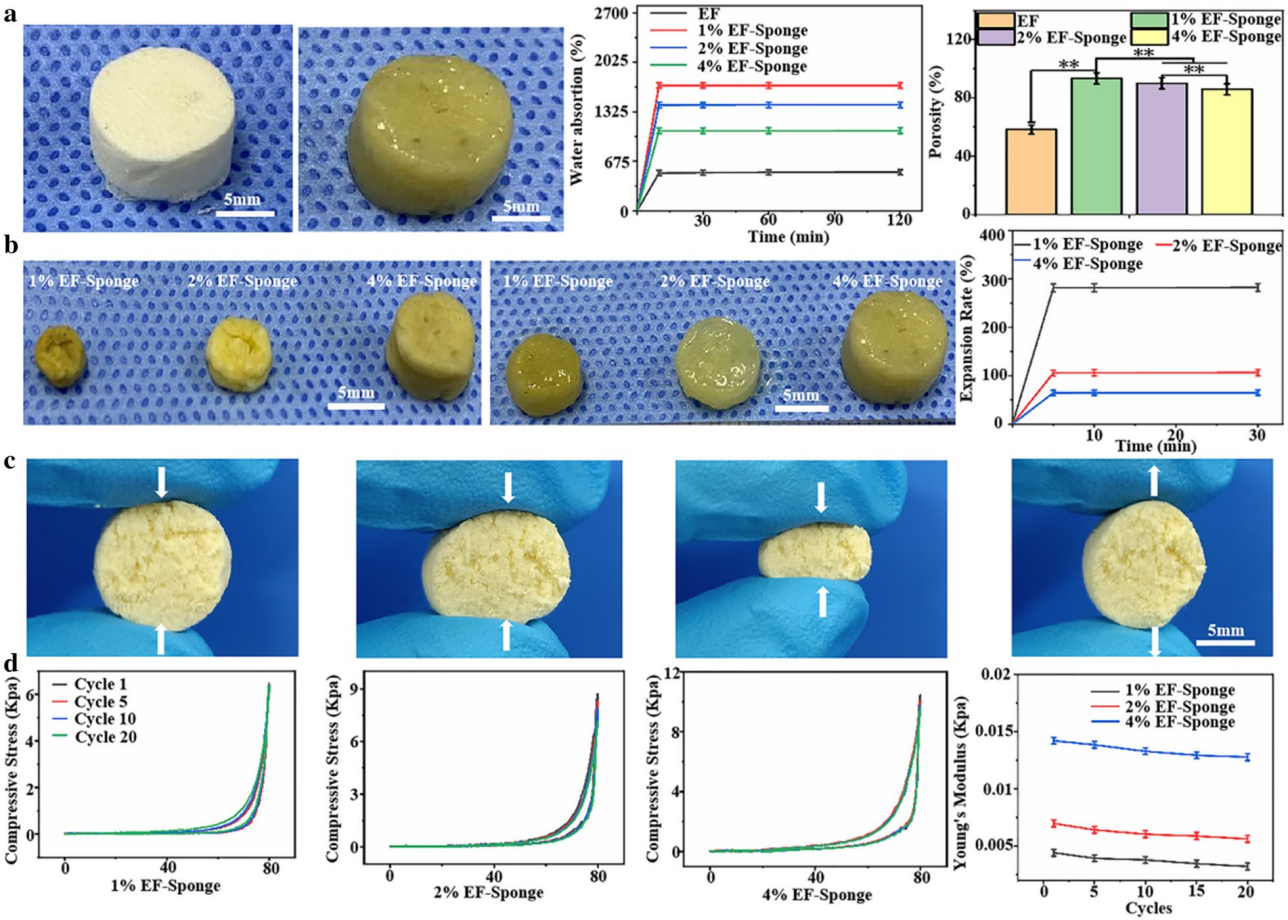
Characterization of EF-Sponges. **a** Analysis of water absorption and porosity of EF and EF-sponges with different fiber densities; **b** Expansion rates of EF-Sponges with different fiber densities; **c** Elasticity photographs of EF-Sponge; **d** Compressive mechanical properties of EF-sponges with different fiber densities

Wettability of biomaterials is one of the most important factors for cells growth and tissue regeneration[26]. In addition, higher water absorption of scaffold would benefit for skin regeneration. After immersion in water, the EF-sponge was observed to rapidly absorb water (Additional file 3: Video 3). The water absorption behaviors of the EF and EF-Sponge were illustrated in Fig. 2a. The maximum water absorption rate of all samples was reached within 10 min. In contrast to the EF sample, the water absorption capacity of the EF-sponge notably varied with fiber density. The increase in water absorption was primarily attributed to the high porosity of the scaffold, in particular, the lower the fiber density, the higher the porosity, and the better the water absorption. The scaffolds with 1% fiber density exhibited the optimal expansion performance (281%), followed by the scaffolds with 2% (106%) and 4% (65.5%) fiber density (Fig. 2b).

The 3D scaffolds exhibited excellent water absorption performance, which is mainly attributed to the porous structure of the scaffolds and the hydrophilicity of gelatin, which is the basic material that comprised the scaffolds. The interconnected porous structure was conducive to the absorption and retention of water. Furthermore, even after several consecutive compressions, the scaffolds still "remembered" their original shapes and quickly recovered it after absorbing water, which may be related to the elasticity and water absorption of the scaffold (Fig. 2c and Additional file 2: Video 2). The enhanced water absorption features of the scaffolds are highly beneficial for biomedical applications. For example, the scaffolds used for skin defect should exhibit good water absorption performance. Wound exudate is a liquid component that leaks from the capillaries due to inflammation that causes the permeability of the capillaries to increase [27]. Too much exudate can cause bacterial infection, and too little exudate is not conducive to cell proliferation [28]. And the shape of skin defect might be various. Therefore, the shape of the scaffolds can be fabricated accordingly and applied to the skin defect. The shaped scaffolds can be perfectly attached to the defect and absorb the exudate from the wound surface to keep the wound moist and prevent infection.

### Mechanical analysis of scaffolds

To evaluate the influence of fiber density on the mechanical properties of the EF-sponge, scaffolds with different fiber densities (1, 2, and 4%) were fabricated. As shown in Fig. 2d, the 1% EF-sponge exhibited good compression recovery after 20 consecutive compressions. The maximum stress in the first cycle was 6.46 kPa, and was decreased to 6.38 kPa after 20 cycles. The Young's modulus also demonstrated a similar trend, dropping from 0.004 (1st cycle) to 0.003 kPa (20th cycles). The 2% EF-sponge sample exhibited marginally lower compression recovery; the maximum stress in the first cycle was 8.52 kPa, which decreased to 7.55 kPa after 20 cycles. Accordingly, the Young's modulus decreased from 0.007 to 0.006 kPa. The 4% EF-sponge sample demonstrated the highest compression recovery, with the maximum stress decreasing from 11.06 to 9.41 kPa. Accordingly, its Young's modulus decreased from 0.015 to 0.012 kPa. Although the 1 and 2% scaffolds exhibited a comparable compression recovery performance after 20 cycles, their maximum stresses were lower than those of the 4% scaffold. Particularly, from the first until the 20th cycle of the compression tests, the Young's modulus of both samples decreased by approximately three times than that of the 4% scaffold. A plausible theoretical explanation for this behavior was that the internal fibers of the scaffold stuck together after absorbing water; in particular, the higher the fiber density, the more the number of fibers that stuck together, and thus, the stronger the mechanical properties of the scaffold.

Scaffold materials play a key role in determining the evolution patterns of cell proliferation and tissue regeneration. The comparison of the three sponge scaffolds showed that 1% EF-Sponge exhibited the loosest fiber distribution, but its mechanical property was the worst. Although 4% EF-Sponge exhibited the best mechanical property, its fiber distribution was the densest which was not conducive to cells infiltration. Therefore, based on the results for the scaffolds with different fiber densities discussed above, the 2% EF-sponge scaffold was selected for further evaluation and denoted as EF-Sponge.

### Biocompatibility and angiogenesis of HUVECs

**Fig. 3:**
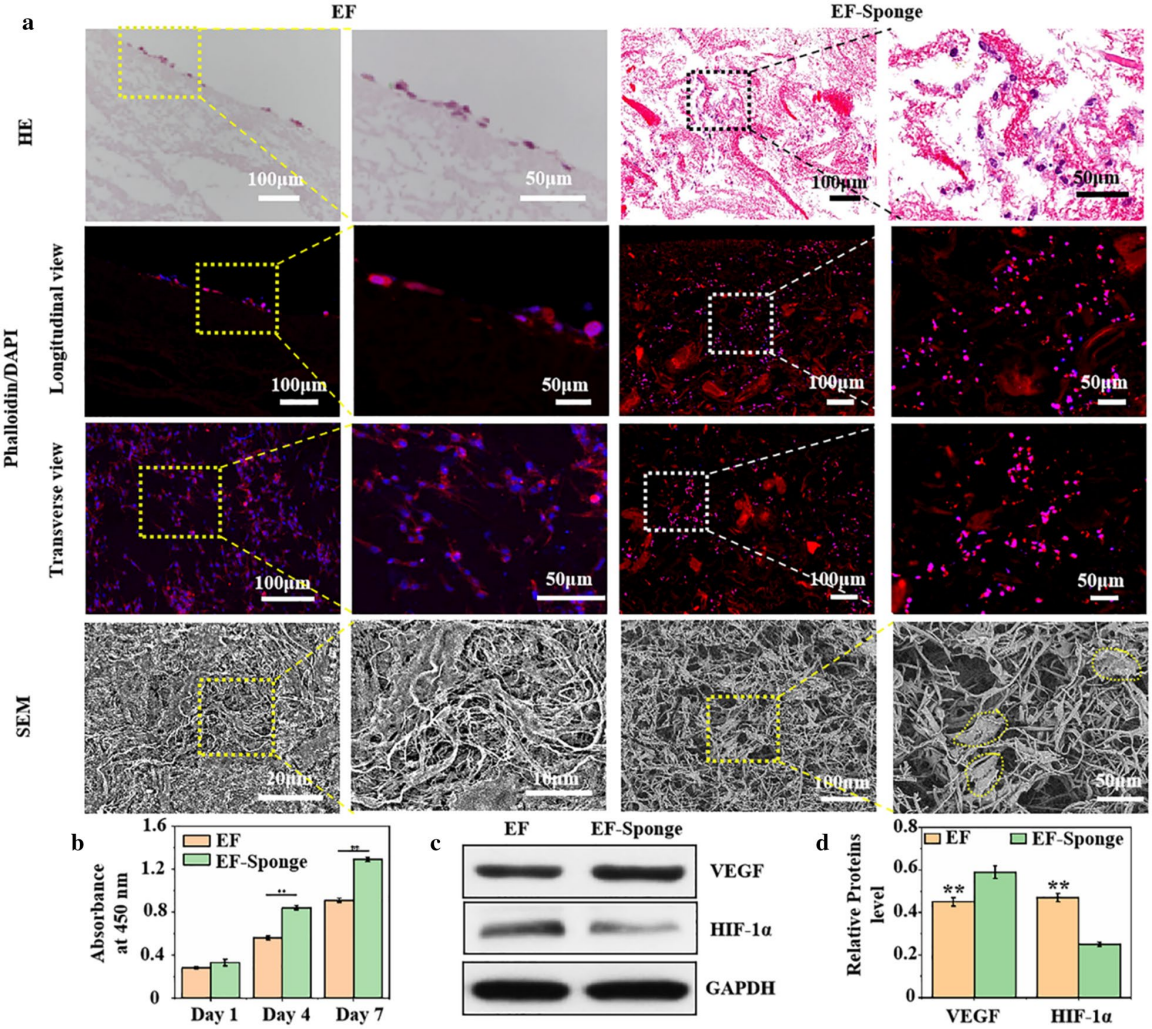
In vitro biocompatibility of HUVECs on EF and EF-sponge scaffolds. **a** Images of HUVECs cultured on EF and EF-sponge, characterized by HE staining, fluorescence staining and SEM; **b** Proliferation of HUVECs cultured on EF or EF-sponge for 1, 4 and 7d (**p < 0.01); **c** Western blotting and **d** semi-quantification of protein levels of VEGF and HIF-1α (**p < 0.01)

To determine the effect of 2D EF membrane and 3D EF-Sponge on cell proliferation, HUVECs were co-cultured with the scaffolds and then tested with the cell counting kit-8 (CCK-8). The experimental results indicated that both the EF and EF-Sponge scaffolds supported the continuous proliferation of HUVECs and there was no significant difference between scaffolds at 1 day. On day 3 and 5, HUVECs showed better proliferation on EF-Sponge than EF (*p* < 0.01) (Fig. 3b). The results revealed that EF-Sponge could promote HUVECs growth and proliferation in comparison with EF.

Figure 3a showed the results for the hematoxylin and eosin (HE) staining, fluorescence staining, and SEM of the HUVECs after culturing on EF and EF-Sponge for 7 days. The cross-sectional fluorescence staining patterns revealed that the HUVECs adhered and grew along the pores of EF-Sponge and were not simply restricted to monolayer adhesion and diffusion as EF. SEM examination also confirmed their ability to attach on the scaffold surface and proliferate along the nanofibers. However, in contrast to EF, the cell cytoskeleton in the EF-Sponge sample extended further in all directions, inducing the nearly direct contact of the cells. The above behavior confirmed that the scaffold structures not only accelerated cell growth and proliferation but also facilitated cell infiltration and three-dimensional distribution.

In addition to proliferation, angiogenesis of HUVECs is crucial for tissue reconstruction. The protein levels of several key markers, such as the hypoxia-inducible factor 1-alpha (HIF-1α) and vascular endothelial growth factor (VEGF), were quantified via western blotting. The results showed that HIF-1α expression in the EF-Sponge was lower than that in EF, whereas the VEGF gene expression demonstrated the same trend (Fig. 3c, d). HIF-1α targets the downstream genes that regulate cell function and angiogenesis under hypoxic conditions [29], therefore, the low expression of this factor in the EF-Sponge indicated that the EF-Sponge sample provided an oxygen-rich environment for the cells, thus stimulating angiogenesis. As such, it was further affirmed that the porous structure of the scaffolds not only promoted cell proliferation but also facilitated the repair process following endothelial injury.

### Evaluation of diabetic wound healing

**Fig. 4:**
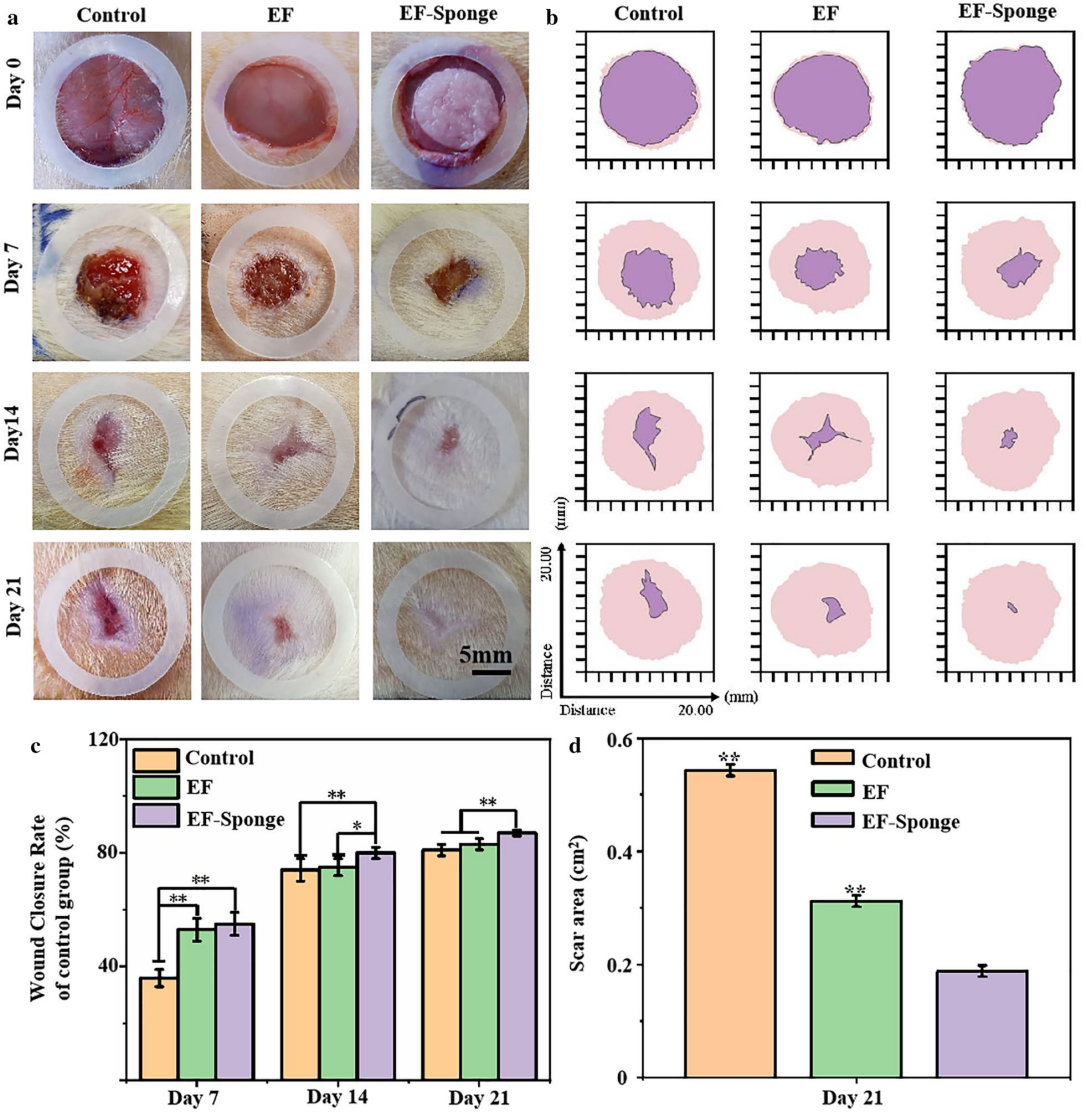
Diabetic wound healing studies in vivo. **a** Images of wound healing with different treatment groups on days 0, 7, 14, 21; **b** Schematic diagram of wound healing in different treatment groups. The pink area meant the initial wound area and purple area represented the wound area at different time points; **c** The wound closure rates at different time points; **d** Quantification of wound area at day 21 (*p < 0.05 and **p < 0.01)

To determine the healing performance of EF-Sponge for skin wound repair, both EF-Sponge and EF were applied to diabetic full-thickness wounds, and the results were compared with those obtained for an untreated control group. The diabetic model was established one week prior to the animal experiments. The blood glucose levels after one week were all above 16.7 mM, with an average value of 30 mM, indicating the successful establishment of the diabetic model. Figure 4a illustrated the wound healing progress in the three groups of diabetic rats at 0, 7, 14, and 21 days after surgery. Gross observation after 7 days revealed that the wound closure area in the control group had expanded at a slow rate, with incomplete scabs and some yellow pus exudation. However, the wound surfaces of the experimental groups were completely crusted and appeared drier and smaller, which was theoretically attributed to the increased hydrophilicity of the experimental materials that absorbed secretions, thus keeping the wound dry [30]. In addition, compared with the EF group, the EF-Sponge group exhibited enhanced water absorption capacity, which further accelerated wound healing. Consistent with the general observation results (Fig. 4b, c), the wound healing rates of both groups were significantly higher than that of the control group (36 ± 3%), reaching 55 ± 4 and 53 ± 4%, respectively. As the treatment time progressed to the 21st day, the wound healing area of the EF-Sponge group had expanded, and a healing rate of 87% ± 1% was achieved, whereas the wound healing rates of the EF and control groups peaked at 83 ± 2 and 81 ± 2%, respectively. Observation of the final effects of the treatments administered to the rats from each group on day 21 showed that the regenerated skin tissues treated with the EF-Sponge had a smaller scar area (Fig. 4d). Thus, the results confirmed that the EF-Sponge effectively promoted the growth of new tissues and reduced the scar tissue area. In summary, our macroscopic observations affirmed that EF-Sponge was beneficial for promoting wound healing.

### Histological analysis

**Fig. 5:**
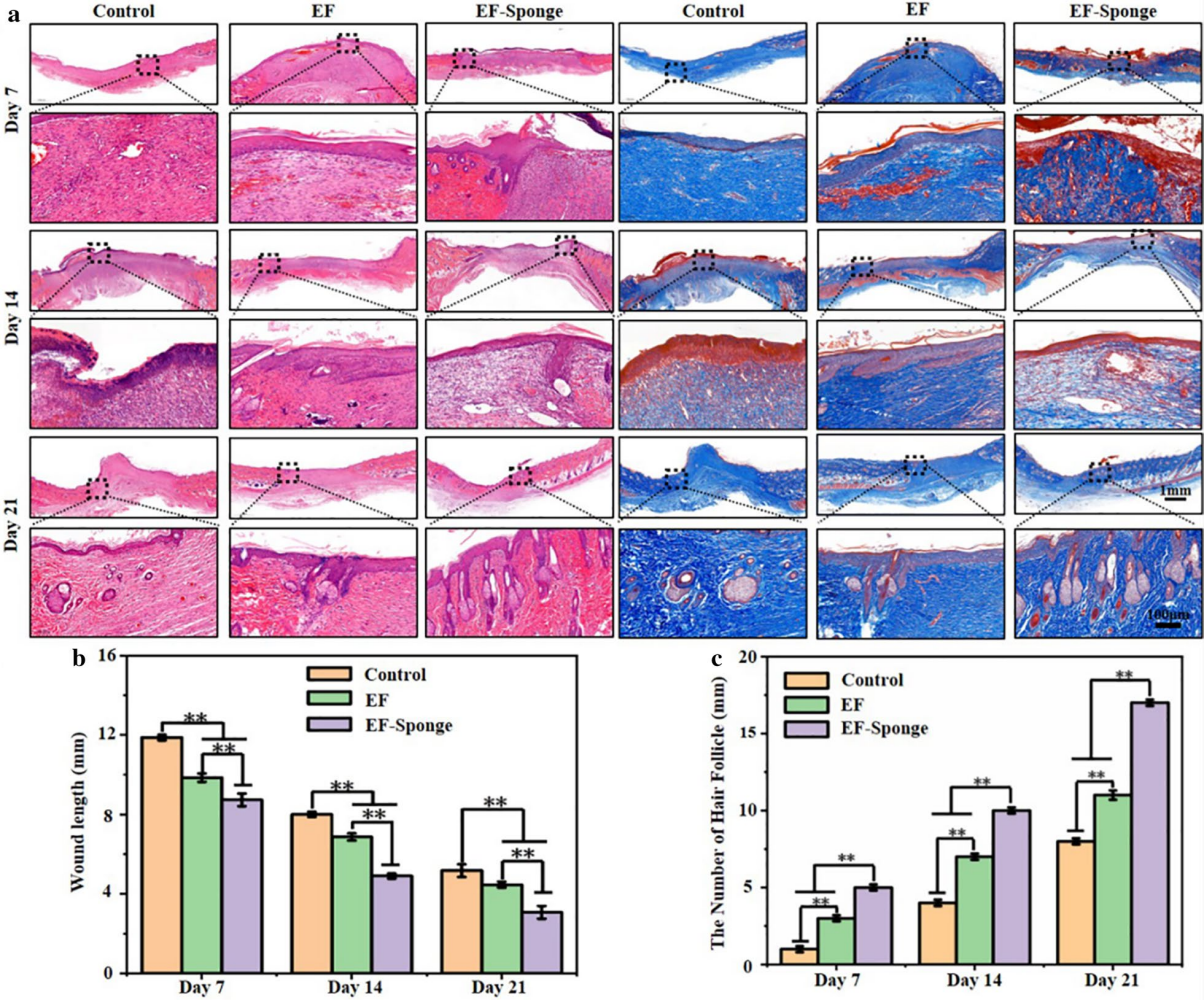
Histological analysis of wound repair in different treatment groups at different time points. **a** HE and Masson staining images of different treatment groups at different time points; **b** Wound healing length and **c** the number of newborn hair follicles in different treatment groups at different time points (*p < 0.05 and **p < 0.01)

The pathology of the wound healing process was assessed by HE and Masson trichrome staining tests (Fig. 5a). Pathological sections from all groups were collected on the 7th, 14th, and 21st day of healing for the tests, the results indicated that the wound length of the EF-Sponge group was significantly shorter than that of the control and the wound length of the EF group was in-between (Fig. 5b). In contrast to the control group, new granulation tissue and epidermis were detected on the wound of the EF-Sponge-treated group, along with tissues resembling skin appendages. These observations suggested that during the early stages of diabetic wound healing, the stimulation of skin appendage formation mitigated the regeneration of scar tissues. Furthermore, the number of new hair follicles in the repaired epithelial tissues was counted (Fig. 5c). The number of new hair follicles in all groups increased with the progress of time, particularly in the EF-Sponge group. The number of new hair follicles in the EF-Sponge was significantly higher than that in the control and EF groups (*p* < 0.01). In summary, histological evaluation of the HE and Masson staining verified the suitability of EF-Sponge treatment for diabetic wound repair based on the acceleration in the formation of granulation tissue and collagen and even skin appendages.

**Fig. 6:**
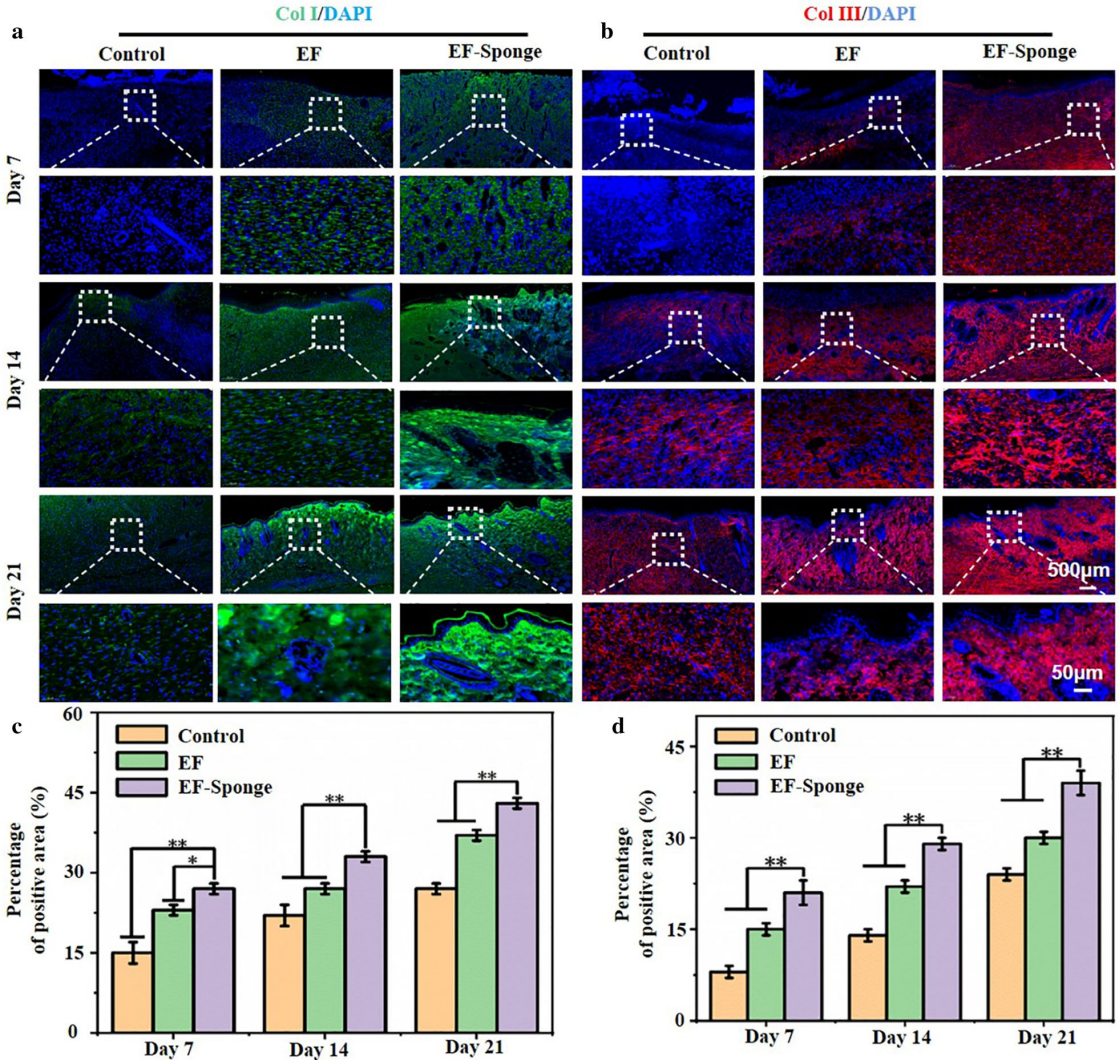
Immunofluorescence staining of collagen deposition. **a** Representative graphs of collagen type I and **b** collagen type III in different treatment groups at different time points; **c** the quantification for relative positive area of collagen type I and **d** collagen type III, (*p < 0.05 and **p < 0.01)

The two main types of skin collagen, type I and type III, are closely related to the mechanism and efficacy of skin injury repair. Figure 6a and c presented the results of collagen I immunostaining. The deposition of collagen I in each group increased with time, and the EF-Sponge group was observed to notably increase. In addition, the expression of collagen III in EF-sponge was significantly increased on the 7th, 14th, and 21st days (Fig. 6b and d). It is well known that the amount of collagen type III of scar tissue is relatively lower than that of normal skin, studies have shown that an adequate amount of collagen type III during the early stages of healing significantly benefits the remodeling process, which leads to less scar tissue formation [31]. By associating the pathological results with the formation of skin appendages, the formation of abundant collagen in the healing process was conducive to the remodeling of collagen matrix, promoting the healing of the wound. Therefore, the treatment of EF-Sponge not only promoted collagen deposition and remodeling, but also played a positive role in inhibiting the formation of scar in the healing tissue.

**Fig. 7:**
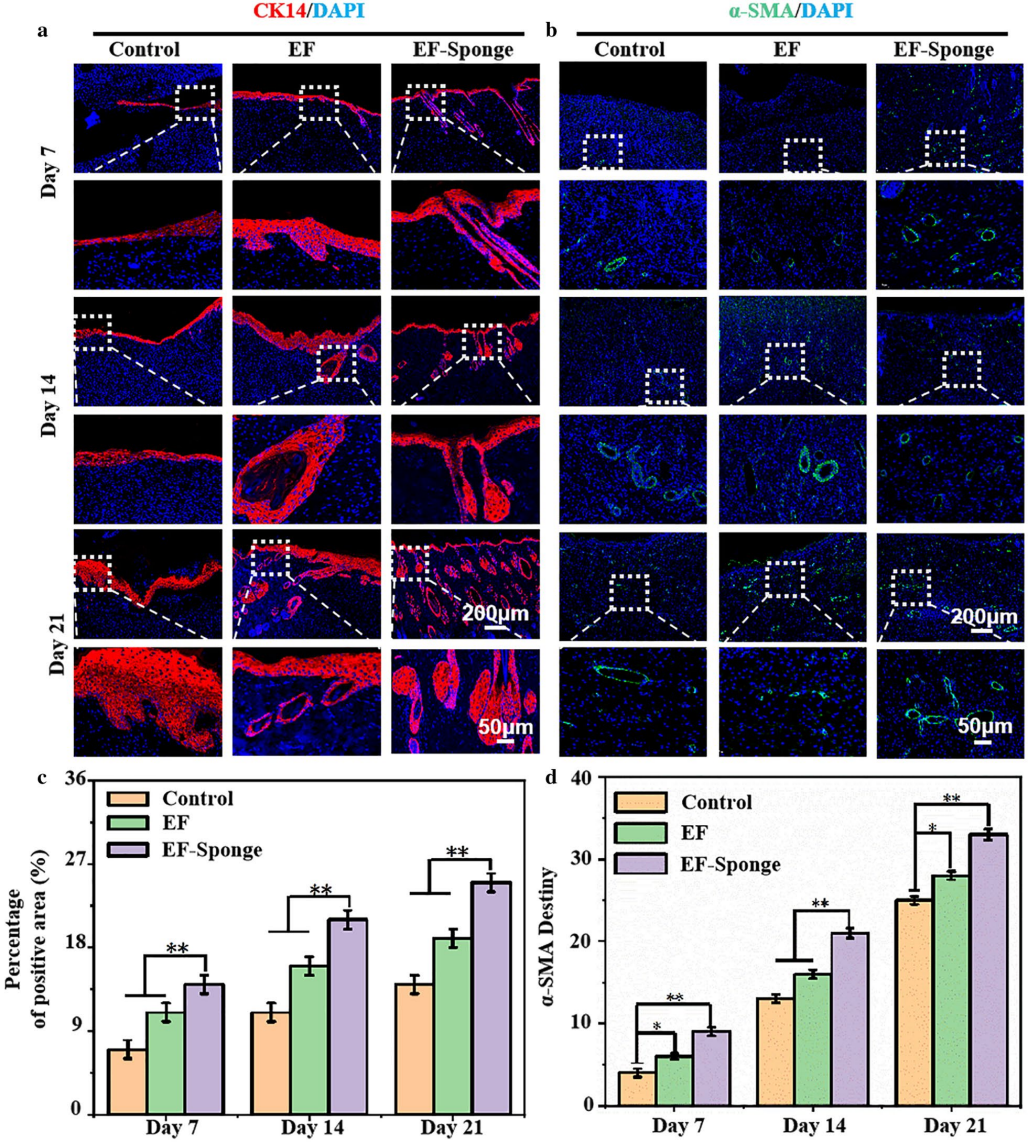
Immunofluorescence staining of cytokeratin and α-SMA in different treatment groups at different time points. **a** Representative graphs of CK14 **b** and α-SMA; **c** the quantification for relative positive area of CK14 and α-SMA, (*p < 0.05 and **p < 0.01)

The expression of keratin and alpha smooth muscle actin (α-SMA) at the repair site was evaluated using immunofluorescence staining. Keratin is an important branch of fibrin that is primarily expressed in the hair and epithelial cells [32, 33]. CK14 is a member of the intermediate fibrin type I keratin family. It can be paired with type II keratin CK5 to form the base keratin of complex squamous epithelial keratin cells, including epidermis and non-exfoliated complex squamous epithelial mucosa [34]. Figure 7a and c presented the results of cytokeratin immunostaining. Except for the control group, the expression of cytokeratin was strongly positive in the two treated samples and especially in the EF-Sponge group. These results confirmed that the process of wound tissue re-epithelialization in both treated groups was enhanced and faster than that in the control group, with the EF-Sponge group exhibiting better healing features than the other groups. On the basis of the optimum behavior of the EF-Sponge group in terms of 3D tissue regeneration, it was speculated that this sample could further promote the regeneration of blood vessels. For this purpose, the vascularization ability of the myofibroblast marker α-SMA was evaluated in vivo at three different time points, by conducting fluorescence expression tests (Fig. 7b, d). Although the number of new vessels was observed to have gradually increased in the control group at all three time points, it was lower than that observed in the experimental groups. The EF-Sponge group demonstrated a significantly higher number of new vessels.

The process of wound repair can be accelerated by enhancing the rate of neovascularization, which in turn provides sufficient nutrients to maintain the newly formed granulation tissue [35]. By promoting the formation of new vessels, the EF-Sponge scaffolds actively contributed to the provision of sufficient nutrients to cells, thereby promoting cell proliferation, which increased the expression of cytokeratin and promoted tissue re-epithelialization. Applying the EF-Sponge in the wound repair treatment process not only stimulated the formation of granulation tissue and new vessels along with collagen remodeling but also contributed to the prevention of scar formation, thus benefiting the proper repair of the diabetic wound.

## Conclusions

In this study, inspired by natural sponge structures, a EF-sponge scaffold with 3D morphologies and ECM biomimetic properties were constructed by a novel and facile method—combination of electrospinning, high-speed homogenization, and thermal crosslinking technologies. In vitro studies indicated that the EF-Sponge could provide a good 3D living environment for HUVECs, facilitating their proliferation, migration, and vascularization. In vivo studies demonstrated that the EF-sponge had a good 3D adhesion to the chronic wound of diabetes in rats. Meanwhile, compared with EFs, the EF-Sponge accelerated angiogenesis and the deposition of collagen and keratin. Thus, the 3D EF-sponge, as biocompatible substrates, has great potential in skin tissue engineering and possibly other biomedical applications.

## Materials and methods

### Materials

Gelatin from porcine skin was provided from Sigma Aldrich. Poly-lactic-acid (PLA) was applied by Jinan Daigang Biomaterial Co., Ltd. (Jinan, China). 1,1,1,3,3,3-Hexaflfluoro-2-propanol (HFIP) was provided by Aladdin industrial Co., Ltd. (Shanghai, China). Tert-butyl alcohol was provided by Rhawn reagent Co., Ltd. (Shanghai, China). Related reagents for cell culture were obtained from Gibco (UK).

### Preparation and characterization of EF-Sponge

Gelatin and PLA were dissolved in HFIP at a mass ratio of 4:1 to prepare gelatin/PLA solution (12 wt%). The optimal electrospinning conditions for gelatin/PLA nanofibers were determined: the applied voltage, the flow rate, and the collection distance were set at 15 kV, 3 ml/h, and 15 cm, respectively. The prepared gelatin/PLA nanofiber meshes were vacuum dried to remove trace solvent. As illustrated in Scheme 1, the preparation of EF-sponge scaffold involved three key steps: (1) cutting of nanofibers into small fragments; (2) dispersion of the small fragments in tert butanol by homogenizing with IKA T-18 at 13,000 rpm for 15 min; (3) frozen in liquid nitrogen for 1 h and then freeze dried for 48 h to obtain the uncrosslinked scaffolds. To stabilize the structure, EF-sponge scaffold was further crosslinked with the thermal crosslinking at 180 ℃ for 2 h in a oven. In addition, EF-Sponges with different fiber densities (1, 2 and 4%) were prepared by varying the weight of the fibers in tert-butanol. The morphology of the fabricated scaffolds was examined by SEM (HITACHI TM-100, Japan). The average nanofiber diameter was measured by Image J Software (National Institutes of Health, USA). The scaffolds were also characterized with a FTIR (Nicolet Nexus 670 FTIR spectrometer).

Water absorption was measured according to the reported method[36]. Each dry scaffold was weighed and recorded (W_d_). The scaffolds were immersed in deionized water for 10, 30, 60 and 120 min, respectively. Then the wet scaffolds was weighed and recorded (W_w_). The water absorption capacity (WA) of the scaffolds was calculated using the following equations:

1 $$WA\left( \% \right) = \left( {{W_w} - {W_d}} \right)/{W_d} \times 100\%$$

The porosity of EF and EF-sponge was evaluated by liquid displacement [37]. Briefly, every scaffold was weighed and measured in dry condition, then soaked in absolute acetone for 48 h. The average porosity was calculated as previously reported [37].

The mechanical properties of the scaffolds were measured by compression cycle test with material testing machine (HY-940FS, China). The compression strain–stress curves of the scaffolds in wet state with compression strain (ε = 80%) were measured at strain rate of 10 mm/min.

### Cell experiments

HUVECs were obtained from the commercial approach and used in vitro experiments, which were incubated in in high glucose medium (Gibco, USA) supplemented with 10% fetal bovine serum (Gibco, USA) and 1% penicillin–streptomycin solution (Hyclone, USA) and incubated at 37 °C in a humidified atmosphere contained 5% CO_2_.

### HUVECs proliferation and migration assessment

HUVECs (1 × 10^4^ cells/well) were seeded on the scaffolds. After 1, 4, and 7 days of incubation, cell proliferation was determined by CCK-8 reagent (Beyotime, China). The morphology of HUVECs on the scaffolds after 7-day culture was examined by HE (H&E) staining staining, immunofluorescence staining and SEM. Briefly, 4% paraformaldehyde was used to fix HUVECs, and dehydrated in a series of ethanol solution. Some dried samples were examined by SEM. The remaining samples were paraffin-embedded and then transected into thin slices to be stained with HE and fluorescence, respectively.

### Western blotting

Protein levels of HIF-1α and VEGF were detected by western blotting as previously reported[38]. The HUVECs cultured for 7 days were treated with trypsin and RIPA lysis buffer, respectively, and their protein concentration was quantified by a BCA protein assay kit (ServiceBio, G2026, China). Next, sample proteins were subjected to 10% SDS electrophoresis followed by transferring onto a PVDF membrane (PVDF, Millipore, US). The membrane was blocked with 5% skim milk for 1 h and then incubated with VEGF (GB11034B, servicebio), HIF-1α (GB11031, servicebio), GAPDH (60,004–1, PTG) antibody, respectively. After the secondary antibodies incubation for 30 min, the blots were visualized by the enhanced chemiluminescence detection system.

### In vivo diabetic wound repair evaluation

All protocols for in vivo experiments were performed in accordance to Institutional Animal Care guidelines. Shanghai Jiaotong University Animal Study Committee approved all procedures involved in animal experiments. 180-210 g male Sprague–Dawley (SD) rats were injected with streptozotocin (STZ) (55 mg/kg, i.p.) to rapidly induce the model of type 1 diabetes by fasting for 8 h before surgery [39]. A week later, the blood glucose level reached above 16.7 mM by measuring the caudal vein of the rats. Rats were randomly assigned to each of the 3 groups (EF, EF-Sponge, and Control), respectively.Two round full-thickness cutaneous wounds (φ = 16 mm) were created on the back of rats. 3 M Tegaderm Film was used to covered the treated wounds to prevent infection. A digital camera was used to recorded the changes of wound repair at day 0, 7, 14 and 21, respectively. The following formula was used to evaluate the wound closure rates (CR):

2 $$CR\left( \% \right) = \left( {{A_0} - {A_c}} \right)/{A_0} \times 100\%$$

A_0_ was the wound closure area at day 0, and A_c_ was the wound closure area at certain day.

HE and Masson’s trichrome staining were used to evaluate the infected wound healing while the immunofluorescence staining such as collagen I and III, cytokeratin, and α-SMA was used to evaluate the collagen deposition, epithelialization, and angiogenesis.

### Statistical analysis

All data were presented as mean ± standard deviation. One-way analysis of variance (ANOVA) was used for statistical analysis to assess the significant differences among the groups. (*) *p* < 0.05 and (**) *p* < 0.01 were considered statistically significant.

## Supplementary Information

**Additional file 1.** The stability of the scaffolds under tense mechanical agitation.

**Additional file 2.** The shape-memory functionality of the scaffolds under the externally applied forces.

**Additional file 3.** The water absorption performance of the EF-Sponge scaffolds.

## Data Availability

All data generated or analysed during this study are included in this article.

## Abbreviations

ECM: Extracellular matrix
EFs: Electrospun fibers
PAN: Polyacrylonitrile
PCL: Polycaprolactone
EF-Sponge: Electrospun fibrous sponge
HUVECs: Human umbilical vein endothelial cells
PLA: Polylactic acid
FTIR: Fourier transform infrared
CCK-8: Cell counting kit-8
HE: Hematoxylin and eosin
HIF-1α: Hypoxia-inducible factor 1-alpha
VEGF: Vascular endothelial growth factor
α-SMA: Alpha smooth muscle actin
HFIP: 1,1,1,3,3,3-Hexaflfluoro-2-propanol
STZ: Streptozotocin
